# Concurrent Occurrence of Internal Resorption in the Crown and Root of the Right Maxillary Lateral Incisor: A Case Report

**DOI:** 10.7759/cureus.116297

**Published:** 2026-09-15

**Authors:** Karthik Rajaram Mohan, Ramachandra Reddy Gowda Venkatesha

**Affiliations:** 1 Oral Medicine and Radiology, Vinayaka Mission's Sankarachariyar Dental College, Vinayaka Mission's Research Foundation (DU), Salem, IND

**Keywords:** cone-beam computed tomography (cbct), endodontic treatment, internal resorption, mineral trioxide aggregates, right maxillary lateral incisor

## Abstract

Cone-beam computed tomography (CBCT) plays an indisputable role in the early diagnosis of internal resorption. Internal resorption often remains silent and appears as an incidental finding in CBCT scans. In the present study, a case of concurrent occurrence of internal resorption in both the crown and root of the right maxillary lateral incisor was reported in a 20-year-old male, with etiology linked to trauma and clinical and incidental radiographic features identified by CBCT, and its management.

## Introduction

Internal resorption involves a root canal or pulp chamber. This resorption results in the enlargement of pulp chamber space or root canal at the expense of the tooth's structure. Internal resorption is transient, progressive, or self-limiting [1].

Internal resorption may affect either primary or permanent dentition. It occurs mostly in permanent central incisors, canines, and first and second molars. It is more common in men than women. Signs and symptoms of internal resorption depend on its location and extent. If internal resorption occurs on the root, it remains clinically silent. If it affects the enamel, the tooth appears pinkish clinically. If extensive, it weakens the tooth and leads to fracture. The pulp expands due to inflammation and communicates with the periodontal ligament, resulting in a deep periodontal pocket or infection [1].

The risk factors for internal resorption include trauma, dental caries, chronic pulpitis, pulpotomy, direct or indirect pulp capping with calcium hydroxide, crack on teeth, invagination of enamel, extreme heat, and orthodontic treatment. The inflammatory process from the above risk factors results in the conversion of undifferentiated cells from the pulp into odontoclasts or macrophages, causing dentinal resorption. Internal resorption results from necrotic infected pulp tissue [2].

Usually, internal root resorption (IRR) is asymptomatic and detected incidentally through routine radiographic examination. During active stages of resorption, the tooth is partially vital and reveals symptoms of pulpitis. The tooth appears pinkish due to the color of the granulation tissue filling the resorbed area [2].

IRR rarely occurs in permanent teeth. It requires different radiographic modalities for diagnosis. Cone-beam computed tomography (CBCT) is superior in the diagnosis of internal resorption. CBCT images with a small field of view (FOV) help diagnose even small areas of resorption. The prevalence of internal resorption is about 9.6% among 1086 patients with no history of endodontic treatment and normal periapical region without any pathology [3].

## Case presentation

A 20-year-old male reported to our department for a routine dental screening. The patient's history revealed that he underwent an episode of trauma two years back. The CBCT sagittal section of the right maxillary lateral incisor #12 is presented in Figure 1. Figures 2A-2B display the axial section of the tooth's crown and root.

**Figure 1:**
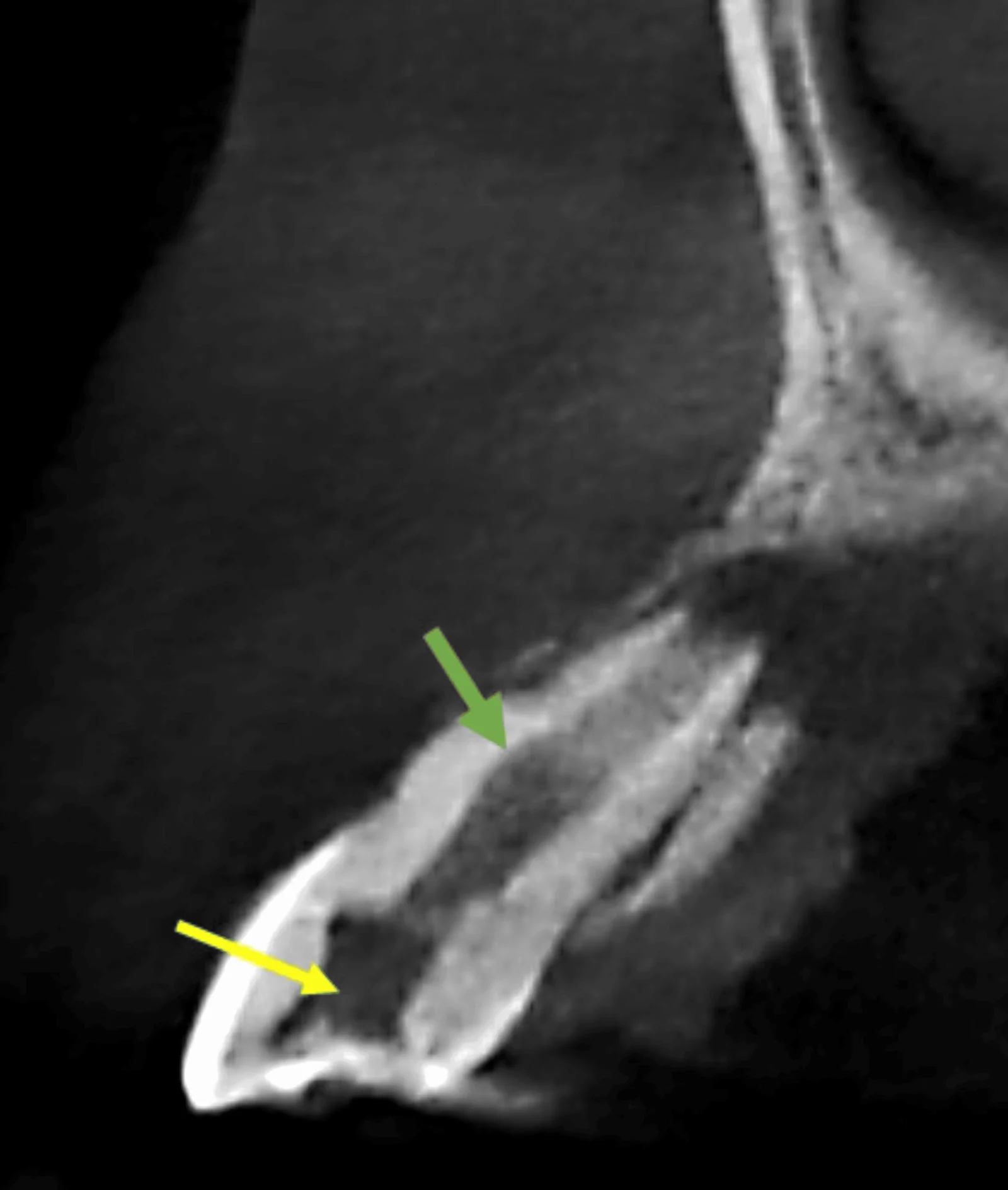
CBCT sagittal section of right maxillary lateral incisor #12 showing enlarged coronal pulp chamber (marked by yellow arrow) and radicular root canal portion (marked by green arrow) CBCT: Cone-beam computed tomography

**Figure 2:**
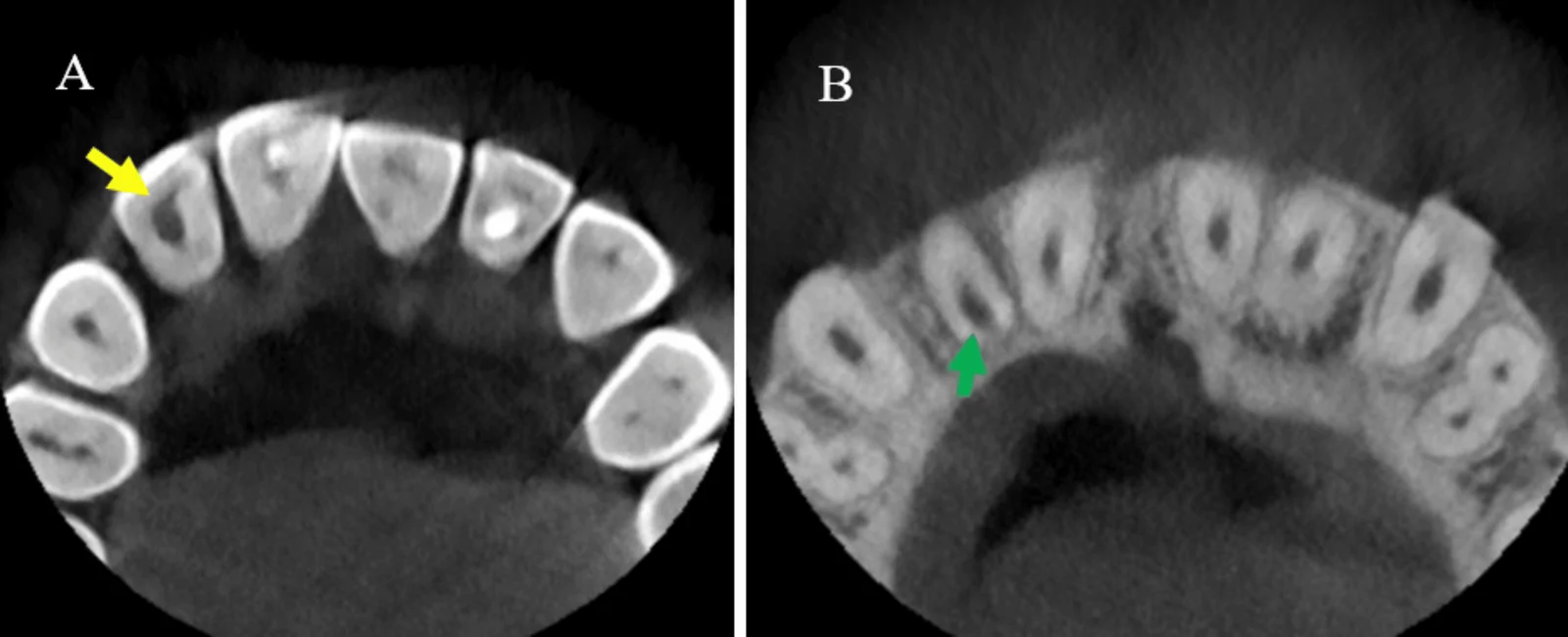
CBCT axial sections of the crown (A) and root (B) of right maxillary lateral incisor #12 showing enlarged pulp chamber in the crown (marked by yellow arrow) and enlarged root canal in the root (marked by green arrow) CBCT: Cone-beam computed tomography

The differential diagnosis includes cervical abrasion or cervical resorption. Cervical abrasion usually has a wedge-shaped defect with a smooth outline. Cervical resorption is an external resorption that appears as radiolucency in the cervical crown portion with pulp involvement and an irregular outline. The area of internal resorption cannot be separated from the root canal space or pulp chamber by varying the horizontal angulation of the X-ray beam. The CBCT sagittal section revealed the presence of a central, round balloon-out-like enlargement of the root canal. The final diagnosis was made as internal resorption based on history and radiographic evaluation by CBCT.

Clinical significance

The case describes a concurrent occurrence of internal resorption in the pulp chamber's coronal portion and the root canal of the right maxillary lateral incisor in a 20-year-old male.

Limitation

Treatment was not done in this case as the patient was asymptomatic and refused treatment.

## Discussion

IRR destroys dentinal tubules and intraradicular dentin due to odontoclastic activities [4]. IRR appears in the radiograph as a symmetrical, oval to round, radiolucent enlargement within the root canal and is ballooned out, clearly defined, smooth, and sharp, with distortion of the outline of the root canal [5]. The pulp chamber or root canal outline has a uniform density. A major difficulty with intraoral radiographs in diagnosing internal resorption is that they only reveal limited diagnostic information because the three-dimensional anatomy of the tooth area being radiographed is projected as a shadowgraph or two-dimensional image in a periapical radiograph [6]. The sensitivity, specificity, sensitivity, and negative and positive predictive values of CBCT images were 76%, 88%, 87%, and 79%, respectively, compared to periapical radiography [7].

Lima et al. stated that CBCT was superior in diagnosing internal resorption in endodontically treated teeth compared to periapical radiographs [8]. Rabinowitch revealed a case of internal resorption in a patient with bruxism and no other history [9]. Kalendar et al. reported a case of true idiopathic internal resorption in multiple molar teeth, including erupted and unerupted molar teeth, with no history of bruxism, periodontal disease, trauma, orthodontic, or bleaching [10]. Urban and Mincik reported a case of internal resorption in a maxillary central incisor and related the cause to interleukin-1 (IL-1) genetic polymorphism [11]. Yoneda reported a case of multiple perforating defects caused by internal resorption associated with oral malodor [12]. Benenati recommended using calcium hydroxide to remineralize defects caused by internal resorption [13]. Meire and De Moor advocated using mineral trioxide aggregate (MTA) to manage internal resorption [14].

The treatment of internal resorption depends on its extent onto the tooth surface. Endodontic root canal therapy halts the resorptive process since root canal treatment deprives the blood supply to clastic cells such as macrophages, odontoblast cells, and granulation tissue. If the internal resorption is extensive, dental extraction is recommended. The dental materials for treating IRR include MTA, resin-modified glass ionomer cement, and thermoplasticized gutta-percha by condensation or injection techniques. The choice of materials depends on whether internal resorption is associated with or without root perforation. MTA is most commonly used for IRR with perforation because of its biocompatibility, potential induction of cementogenesis and osteogenesis, and sealing ability. For internal resorption without perforation, gutta-percha with thermoplasticized gutta-percha obturation technique is recommended.

## Conclusions

CBCT is indisputable in the early detection and diagnosis of internal resorption. Internal resorption is usually asymptomatic and is often diagnosed as an incidental finding in radiographic examination. Undoubtedly, CBCT is a better radiological diagnostic aid in diagnosing internal resorption.
